# Indigenous knowledge of HIV/AIDS among High School students in Namibia

**DOI:** 10.1186/1746-4269-7-17

**Published:** 2011-06-09

**Authors:** Kazhila C Chinsembu, Cornelia N Shimwooshili-Shaimemanya, Choshi D Kasanda, Donovan Zealand

**Affiliations:** 1 Department of Biological Sciences, Faculty of Science, University of Namibia, P/B 13301, Windhoek, Namibia; 2 Department of Science, Mathematics and Sports Education, Faculty of Education, University of Namibia, P/B 13301, Windhoek, Namibia

## Abstract

**Background:**

The use of Indigenous Knowledge (IK) can help students to form schemas for interpreting local phenomena through the prism of what they already know. The formation of schemas related to HIV/AIDS risk perception and prevention is important for individuals to form local meanings of the HIV/AIDS epidemic. The objective of this study was to explore the indigenous names and symptoms of HIV/AIDS among High School students in Namibia

**Methods:**

Focus group discussions were used to collect qualitative data on indigenous names and symptoms of HIV/AIDS from students in 18 secondary schools located in six education regions. Data were grouped into themes.

**Results:**

People living with HIV/AIDS were called names meaning prostitute: *ihule, butuku bwa sihule*, and *shikumbu*. Names such as*kibutu bwa masapo *(bone disease),*katjumba *(a young child),*kakithi *(disease), and*shinangele *(very thin person) were used to describe AIDS. Derogatory names like *mbwa *(dog), *esingahogo *(pretender), *ekifi *(disease), and *shinyakwi noyana *(useless person) were also used. Other terms connoted death (*zeguru*, heaven; *omudimba*, corpse), fear (*simbandembande*, fish eagle;* katanga kamufifi*, (hot ball), and subtle meaning using slang words such as 4 × 4, *oondanda ne *(four letters), desert soul, and mapilelo (an AIDS service organization). Typical (body wasting) and non-typical (big head, red eyes) symptoms of HIV were also revealed.

**Conclusions:**

The study determined students' IK of the names and symptoms of HIV/AIDS. Programmes to prevent/manage adolescent HIV infection and stigma may be strengthened if they take students' indigenous understandings of the disease on board.

## Background

Indigenous Knowledge (IK) is an important foundation for sustainable and innovative solutions in education, health, agriculture, and biotechnology. At a regional symposium in South Africa, Nkondo cautioned that the quest to understand and use Indigenous Knowledge Systems (IKS) should not be likened to "primitive anthropology" [1]. According to Nkondo, IK has a clear link between thinking and action, theory and practice, and mind and body [1]. Nkondo [1] and Teffo [2] argued that African IK adequately fits into the two epistemological denominations of rationalism and empiricism. They maintained that African IKS were not static. On the contrary, African IKS were situation-dependent, continuously-evolving, and actively adapting to the ever changing world [1,2]. Be that as it may, African research and educational institutions have now reinvigorated efforts to interface and mainstream IKS into their programmes. In South Africa, the Department of Science and Technology has positioned IKS at the core of their vision and blueprint for scientific development and innovation [3].

In terms of the school curriculum, the use of IKS can help students to form schemas for interpreting local phenomena through the prism of what they already know [4,5]. It has been postulated that all human beings possess categorical rules or scripts that they use to interpret the world [4,5]. New information is processed according to these rules, called schema [5,6]. The schema theory views organized knowledge as an elaborate network of abstract mental structures which represent one's understanding of the world. Therefore, schema theorists insist that prior knowledge is an important starting-point for effective learning and instruction [5,6].

Cultural factors are important in health-related schemas [7], and there are suggestions that narratives of illness are embedded in a unique set of life circumstances and guided by individual schemas and explanatory models [8]. Therefore, in order for students to successfully process new information about HIV/AIDS, their indigenous schemas which are related to the new content must be activated [6]. Thus IK is an important cog for the formation of schemas related to HIV/AIDS risk perception and prevention. IK helps individuals to form social constructions and local meanings of the HIV/AIDS epidemic as supported by the interpretative paradigm. This paradigm posits that subjectively-based reality is influenced by culture and history. Since HIV/AIDS is believed to have originated from Africa [9], it is natural that a substantial amount of IK about HIV/AIDS does exist. Thus, people make sense of HIV/AIDS through their own cultural beliefs, historical narratives, and indigenous understandings.

Several studies have documented the indigenous understandings of HIV/AIDS among traditional healers in Zimbabwe [10] and school managers in South Africa [11]. Various categories of meaning of HIV/AIDS have been revealed: biomedical, cultural, religious, witchcraft, race, and eschatology [11]. It was noted that indigenous beliefs have a measurable association with attitudes to HIV/AIDS prevention [12]. Specifically, biomedical and traditional views about prevention were found to be in direct conflict with one another, and this undermines the likelihood of people to take precautions against HIV/AIDS [13]. For example, since the transfer of semen is culturally considered to be important for optimal foetal development during pregnancy [14], it was difficult to change attitudes against unprotected sex between an HIV-positive husband and a pregnant wife that was HIV-negative.

In Namibia, the first four cases of HIV/AIDS were diagnosed in 1986 [15]. Therefore, for over two decades, indigenous people have witnessed the impacts of HIV/AIDS in their households and neighbourhoods. Through close interaction with relatives or neighbours that are infected with HIV/AIDS, people have accrued a lot of IK about HIV/AIDS. Although such IK may not be scientifically verified, local communities still use it in their informal discussions of and behavioural interventions against HIV/AIDS. Rompel [16] documented that in Oshiwambo, HIV is called *omukithi gwonena *which means modern disease or developmental disease. That label means that HIV/AIDS is deeply embedded into modern living conditions; that AIDS has a lot to do with modernity. AIDS is also called "the disease" or "the three-letter-illness", and the terms "HIV" and "AIDS" were rarely used [16].

Therefore, it is important that the formal school HIV/AIDS curriculum is implemented within a microcosm of IK of HIV/AIDS. Thus, formal HIV/AIDS education should take into account the indigenous jargon of HIV/AIDS that people use. In fact, UNESCO [17] also recommended that HIV/AIDS curricula should acknowledge the prior knowledge, experiences, and obstacles of the students. This can help to dispel some of the stigma and widely held myths or misconceptions about HIV/AIDS. Critical theory also implores the secondary school HIV/AIDS curriculum to promote in students an awareness of themselves as social beings [18]. It demands that the language used in the teaching and learning of HIV/AIDS should be that of teachers and students, from their everyday lives and contexts; language that helps individuals to discern their daily social interactions with HIV/AIDS.

In Namibia, IK about HIV/AIDS has not been mainstreamed into HIV/AIDS education and interventions. In secondary schools, HIV/AIDS education is delivered through science subjects such as Life Science (for students in Grades 8-10) and Biology (for students in Grades 11-12). In Grade 9 Life Science, HIV/AIDS is taught during the topic on health education. Here, the HIV/AIDS content is restricted to types of HIV tests, knowing one's HIV status, symptoms of HIV, and statistics of the global epidemiology of HIV/AIDS in 2001 [19]. In the Grades 11-12 Ordinary level Biology syllabus, the role of a balanced diet for HIV-positive persons is taught under the topic on nutrition in humans [20]. The topic on human reproductive system contains further content on HIV/AIDS, namely: methods of transmission and prevention, increased vulnerability of Namibians to other illnesses due to the increased prevalence of HIV, and the socio-economic consequences of AIDS [20]. Preventive interventions are delivered through *My Future is My Choice*, a UNICEF-sponsored programme which emphasizes the ABC (Abstinence, Be faithful, and use Condoms) approach [21].

Given the importance of IKS to the formation of schemas, social meanings, and subjective realities of HIV/AIDS, we hypothesized that the teaching and learning of HIV/AIDS may be enriched by the inclusion of students' IK into the secondary school HIV/AIDS education curriculum. To our knowledge, uncovering indigenous understandings of HIV/AIDS has not been done among High School students. Thus, the objective of this study was to explore the indigenous names and symptoms of HIV/AIDS among High School students in six regions of Namibia.

## Methods

### Ethical approval

Ethical permission to conduct the research was obtained from the University of Namibia Post-graduate Studies Committee. Permission to conduct the research in secondary schools was sought from the Permanent Secretary of the Ministry of Education in Windhoek. In the regions, permission to visit the schools was received from the Regional Directors of Education. At the schools, permission was obtained from the school principals, and students were informed that they were free not to participate in the study.

### Data collection and analysis

Data were collected between October and November 2009. A cross-sectional survey involving a three-stage sampling design was utilized. A cross-sectional design was appropriate because it was a snap-shot exploration that allowed a statistically significant sample of a population to be used in estimating the relationship between an outcome of interest and population variables as they existed at a particular time. The primary sample included six education regions: Caprivi, Kavango, Ohangwena, Omusati, Oshikoto, and Khomas. The regions were purposefully selected because of their high prevalence of HIV/AIDS (> 15.0%). Eighteen government-run secondary schools (three from each region) were randomly selected into the secondary sample. Within the schools, data were collected from randomly selected classes of either Life Science or Biology students (the tertiary sampling units). The Life Science and Biology students were included into the sample because they studied HIV/AIDS in these subjects. Teachers were requested to leave the classroom immediately after the researchers were introduced to the students. This helped to reduce the intimidation of the students. After obtaining their verbal consent, the class of students was divided into two groups.

The data gathering phase involved collection of demographic data and focus group discussions. There were a total of 829 students in the 36 focus groups. The students' ages ranged between 13-27 years with a median age of 17 years. They hailed from various ethnic groups: 62.0% were Ovambo, 14.8% were Kavango, 3.5% were Herero, 3.6% were Damara-Nama, and 2.3% were classified as 'others' (Basters, Tswanas, Afrikaans, and non-Namibian nationals). There were 44.1% male and 55.9% female students. Focus groups consisted of both male and female students.

Focus groups were allowed to discuss various local names and symptoms that people in their communities associated with HIV/AIDS. The discussions were led by the researchers. The two standard questions in the focus groups were: "what names do people associate with HIV/AIDS?" and "what symptoms do people associate with HIV/AIDS?" Qualitative data were recorded into note books and indigenous terms were later translated into English by experienced local translators. Names associated with HIV/AIDS were grouped into the following themes: sex, prostitutes, HIV infection, AIDS syndrome, fear-factor, derogatory names, witchcraft and slang. Symptoms of HIV/AIDS were divided into two themes: typical symptoms and non-typical symptoms. Cross-checking of data was done in order to determine predominant terms for names and symptoms of HIV/AIDS.

## Results

The names that people associated with HIV/AIDS are presented in Table 1. In the Caprivi region, HIV/AIDS was commonly referred to as *simbandembande *which is the name of the fish eagle in the indigenous Lozi language. People suffering from HIV/AIDS were also called *mapilelo*, a name of a local Non-Governmental Organization (NGO) that provides home-based care for people living with HIV/AIDS. In the Kavango region, people living with HIV/AIDS were called *ihule *(or *sikumbu*) which mean prostitute, *esingahogo *(means a pretender or a snake), and *zamu zuguma *(which means victim).

**Table 1:** Indigenous names associated with HIV/AIDS and their putative English translations in different regions

Regions	Local names for HIV/AIDS (English translation)
**Caprivi**	Prostitutes: *Butuku bwa sihule *(disease for prostitutes); m*bushahi, ndarabangwa, buhure *(promiscuous) AIDS syndrome: *Ci lwala *AIDS (suffering from AIDS); *Kibutu bwa masapo *(bone disease); disease without cure Derogatory names: *Mbwa *(dog); i*cho *(there he/she goes) Death: Dead girl; Mr. Killer Fear-factor:***Simbandembande *(eagle); killer disease; ***mamuingelele *(disease that takes everything or everyone); m*ashinya bomu *(destroys without mercy) Slang: English; George; k*alikaava *(she hit herself or he hit himself); m*apilelo *(place where people are saved) Witchcraft: *kaliloze *(gun)
**Kavango**	Prostitutes: Whore; **i*hule ***(bitch or prostitute); foolish prostitute; s***ikumbu ***(prostitute) HIV infection: ***Kambumburu ***(HIV virus);***s**akwata kehamba *(got infected by HIV) AIDS syndrome: Bad disease; k*arukukute *(a skinny person); k*atjumba *(a young boy, child); **skeleton** Derogatory names: Chameleon; e*singahogo *(pretender, snake, somebody that comes up with bad ideas in which they do not take part); *kangweru *(a liar); **Mosquito**; transmitter Death: Living corpse; dead-alive; living on borrowed time; z*eguru (keguru *is something of heavenly nature) Fear-factor: Calamity; *hepeka nyoko *(make your mom to suffer); *ngomana (*you are finished); z*amu zuguma *(something has been thrown at him/her; victim) Slang: 4 × 4 (name of a local musical band, a stronger type of vehicle); *CD; *Sida (AIDS in French); English; s*hikembandai *(a bird)
**Khomas**	Prostitutes: Bitch; *Mate* HIV infection: ***Ekiya ***(thorn);*Ombuto *(HIV virus) AIDS syndrome: AIDS boy; Bones; ***Ekomba ***(AIDS); Killer disease Derogatory names: rotten apple; Red house; Donkey; *Ekifi *(disease); **Fool;***Gaba xub/xus *(go and die you bastard); Mr/Mrs AIDS, HIV, Skinny, or Skeleton Death: Dead; killer Slang: **Four letters**(HIV or AIDS)
**Ohangwena**	Sex: *Okapendi *(underwear); sugar-dad HIV infection: ***Ekiya ***or ***Okakiya ***(thorn); **Infected people;***Otalumbu nombuto *(living with the virus); ***Owayapa ***(HIV-infected) AIDS syndrome:**Killer disease** Death: Dead baboon; dead body; o*mudimba *(corpse); *Oto kunghula nombila, etsetse *(approaching death); dead-alive Fear-factor: Fire; *Katanga kamufifi *(hot ball); person on the red line; lion; victims Slang: **Four letters **(HIV or AIDS); **modern disease**
**Omusati**	Sex: *Ekululume *('real man') Prostitute:*Shikumbu *(bitch) HIV infection: *aantu yena omukithi gwoshinanena *(HIV/AIDS infected people); **Infected people; ***ekiya, Kakiya *(small thorn); *namukithi ta kunu ombuto *(person spreading the virus); *ombuto, Omuntu talumbu nombuto (virus/infected person); okuyina *(already infected); *okwayapa, yapa *(somebody that is already infected) AIDS syndrome:*Oshimbebe *(very weak); *oshinkapa *(very weak or disabled); ***Osuvi ***(AIDS); *aantu mboka yeli kepango *(people on ARVs); **AIDS people; **ARVs; *idisa *(disease); *omukithi, kakithi *(disease); *masipa *(bones); *kuundanda une *(of 4 letters or AIDS); *obustanga *(AIDS); *odjou *(AIDS); **patient; **uncured disease Derogatory names: Idiot; *kaavulika *(somebody who does not listen to advice or is stubborn) Death: ***Kadhipagi, dhipagi, edhipagi-kithi ***(killer disease); *nakusa, onakusa *(diseased, someone who will die); dead body Slang: **Four letters **(HIV or AIDS); Mr. Four letters; Desert soul; *skele *(skeleton); waiter; Mr. Deeds Witchcraft: *omulodi *(witch)
**Oshikoto**	Sex: *aaholiyiipala *(people addicted to sex); *akulyuunona *("child killer" or sex); *ekululume *('real man'); *tondo *(testes) HIV infection: Carrier; *ota yapa *(he or she is caught or HIV-infected); ***okakwega ***(small thorn); **positive people** AIDS syndrome: A bag of bones; **AIDS people, infected people, infections; ***omukwati gwepango *(somebody receiving treatment); *shinangele *(very thin person); stick Derogatory names: *Shinyakwi noyana *(somebody useless with his/her children) Death: Only one month ahead Fear-factor: *Iihakanwa *(AIDS victims); disaster; terminator Slang: **Four letters **(HIV or AIDS); ***Oondanda ne ***(four letters or AIDS); skeleton

Terms in bold were cited several times

In the Khomas region, AIDS was often called *four letters *or *ekifi *(meaning disease). In the northern regions of Ohangwena, Omusati, and Oshikoto, HIV/AIDS was generally called *ekiya *(or *okakiya*), which in the indigenous Oshiwambo language means thorn. Other Oshiwambo references to HIV/AIDS included *kadhipagi *(killer disease), *aaholiyiipala *(people addicted to sex), *nakusa *(someone who is about to die), *okakwega *(small thorn), *osuvi *(AIDS), *akulyuunona *("child killer" or sex), and *kaavulika *(someone that does not listen to advice).

The symptoms associated with HIV/AIDS are listed in Table 2. The typical symptoms of HIV/AIDS were weight loss, flu, fever, diarrhoea, coughing, and swollen glands. Non-typical symptoms of HIV/AIDS are also listed in Table 2. They included red lips, impaired vision, red eyes, big head, small pox, unfriendliness, painful joints, change in body colour, stiff neck, high blood pressure, dizziness, and loss of hair.

**Table 2:** Symptoms associated with HIV/AIDS in different regions

Regions	Typical symptoms	Non-typical symptoms
**Caprivi**	**Weight loss**, **flu **or colds, **fever**, **diarrhoea**, **vomiting**, **coughing**, neck pain, thin, getting thin without being sick, high temperature, gonorrhoea, syphilis, **TB**, headache, tiredness, **loss of appetite**, malaria, night sweats, **weakness**, pain when passing urine, rash, sores on body, spots on body, stressed, lonely, **tired**, and pneumonia.	Body changes colour, cannot work, red lips, impaired vision, aloof, painful joints, and **hair changes colour**.
**Kavango**	Burning during urination, **coughing**, **diarrhoea**, **weight loss**, fatigue, headache, fever, having different diseases, high temperature, insanity, blind, TB, loss of appetite, loss of weight and body colour, low CD4 count, many opportunistic infections, persistent dry cough, sores on body, sores on sex organs, sores around anus, **skin rashes**, **tired **and weak all the time, and vomiting.	Dizziness, too fat, spots on face, and pimples.
**Khomas**	**Thin**, coughing, **fever**, vomiting, nausea, skinny, flu, fragile and sick, gonorrhoea, headaches, helpless, loss of appetite, **loss of weight**, sick every time, skinny, **sores on body**, sores on genitals, **TB**, and **rashes**.	Red eyes, laziness, afraid of being with others, their shape starts to change, sleeps too much, and their stress levels increase.
**Ohangwena**	**Diarrhoea**, **coughing**, high blood pressure, always sick, **thin**, body weakness, headache, change of skin colour, loss of appetite, coughing deep, ulcers around mouth, unexpected weight loss, fever, **body sores**, impaired vision, **loss of appetite**, persistent cough, **whooping cough**, **low CD4 count**, have STDs most times, pimples around body, **skin rashes**, swollen glands, swollen skin, syphilis, gonorrhoea, TB, malaria, **tired**, night sweats, and many opportunistic infections.	**Neck stiffness**, **neck pain**, loses temper, not peaceful, stays away from relatives, swollen legs, and skin becomes dark.
**Omusati**	TB, **weight loss**, **thin**, coughing, many diseases manifest at once, body weakness, **loss of appetite**, persistent cough, headache, **diarrhoea**, bad cough, STDs, fever, tiredness, **body weakness**, high body temperature, sores around mouth, lack of confidence, cannot work long hours, loss of energy, many wounds on body, rashes, syphilis, gonorrhoea, short body, **sores on genitals**, **swollen glands**, **vomiting**, feeling cold, and **weak**.	Big head, change of skin colour, loss of muscles, vomiting, sneezing, running nose, hair becomes yellow and old-like, hair falling, smallpox on face, **sores around mouth**, bald head, red eyes, sore lips, very angry at people, and stressed.
**Oshikoto**	**Coughing**, vomiting, **thin**, **neck stiffness**, **diarrhoea**, **fever**, **loss of appetite**, **headache**, tired, stressed, **weight loss**, low CD4 count, loss of body colour, STDs, TB, and **fatigue**.	Angry, **impaired vision**, neck pain, sneezing, swollen muscles, unfriendly to others, dizziness, and wounds all over the body.

Terms in bold were cited several times

## Discussion

Indigenous names used to refer to HIV/AIDS, at least in part, influence how people perceive their susceptibility to HIV/AIDS. Such names signify how people think about the disease. They also might help or hinder efforts aimed at creating interventions based on indigenous understandings of HIV/AIDS. Through such names and caricatures, individuals interpret and find personal meanings, actions, and behaviours towards HIV/AIDS.

Some of the indigenous names of HIV/AIDS in this study revealed that Namibians think of HIV/AIDS as a condition that affects individuals that love sex, for example prostitutes. In many African countries, irresponsible, immoral and promiscuous sexual behaviours are commonly believed to be responsible for the heterosexual HIV epidemic, regardless of the epidemiological reality [22]. Derogatory names for HIV/AIDS reflected the stigma associated with the disease. Slang labels also highlighted the fear-mongering and subtle warnings towards HIV infection and death. In the Caprivi, the region hardest hit by HIV/AIDS in Namibia, the use of the name *simbandembande *is meant to scare people from engaging in risky sexual behaviours because HIV/AIDS quickly takes away people's lives, much the same way as the fish eagle takes away small fish from the water. Engaging in these discourses therefore fuels the individualistic (micro) and collective (macro) social forces that galvanize preventive sexual behavioural norms in the local community.

Furthermore, people in the Caprivi region that are HIV positive often blame witchcraft as the cause of illness. In this way, HIV/AIDS is likely to be considered a more socially "acceptable" illness narrative [23]. While HIV/AIDS is seen by many to be self-inflicted and therefore preventable, witchcraft is beyond the control of the individual, and blame for the illness is externalized [23]. In many cases, therefore, witchcraft narratives can be seen as an active coping strategy which enables the ill person to receive continued care and sympathy, and permits open discussion of the illness without stigmatizing the household. However, accusations of witchcraft can result in emotional distress, long-term divisions within families, and subsequent loss of key social support networks with adverse implications for livelihood security.

The appellation of HIV/AIDS as *mapilelo*, a local NGO providing home-based care to AIDS patients, invokes a sense of helplessness and dependency that accompanies this debilitating disease. It also helps to shape the attitudes of people that would be infected with HIV/AIDS towards help-seeking. Social stereotypes are a type of role schema [24]. Thus, in terms of the schema theory, the various appellations of HIV/AIDS help individuals to integrate and appreciate the multifaceted complexities of people living with HIV/AIDS. Social stereotypes of people with HIV/AIDS can also be a form of categorization that may lead to HIV risk avoidance. Elsewhere, AIDS metaphors such as death, horror, punishment, guilt, shame, and fears of contagion and disease have reinforced stigmatization and discrimination [22].

On the other hand, local caricatures and social constructions of HIV/AIDS are an important component of the school's hidden curriculum. The hidden curriculum is defined as those unstated norms, values, and beliefs embedded in and transmitted to students [25]. The hidden curriculum is important because it allows teachers and students to grasp HIV/AIDS as a societal phenomenon. It also captures the 'structural silences' that shape the form and content of school knowledge; yet such voices are usually excluded from public discourses and rationales for HIV/AIDS education [25]. Therefore, it is crucial to note that there is always more going on in the school than we realize.

Again, the point is that the study of HIV/AIDS is too culturally-sensitive, subtle and dynamic to completely capture into a formal school terminology and curriculum. To circumvent such shortcomings, public knowledge of the local caricatures of HIV/AIDS, including their street or village lingo, are a powerful form of the hidden curriculum that, if harnessed properly, may transform secondary school teachers' and students' knowledge and perceptions of HIV/AIDS. Due to the silence around HIV/AIDS, the Ovambo people prefer to be subtle when referring to the condition. Thus HIV/AIDS is called *ekiya *(thorn), *katanga kamufifi *(hot ball), and *owayapa *(HIV-infected). These cultural references to HIV become shared learned meanings that are transmitted for the purposes of promoting individual and societal adjustment to the AIDS epidemic. The slang and derogatory appellations to HIV/AIDS also show that the shared meanings are dynamic and subject to continuous modification in response to the changing epidemic.

Selikow [26] asserted that although there is a lot of interest about the unique socio-cultural contexts in which HIV infection occurs, there is scanty evidence about the role of indigenous languages in HIV/AIDS prevention. It was noted that South African youths have a specialist township language that they use to refer to sexuality and HIV/AIDS. Within that prism, language used to describe HIV is reinvented so that healthier sexualities are encouraged [26]. In this limelight, we contend that the cultural silence and taboos associated with AIDS in Namibia are inherent in the language used to describe HIV infection and its related symptoms. This contention is supported by observations that the social construction of AIDS as *omukithi gwonena *is not to be misunderstood as a backlash to Europeans because they have accused Africans to be the source of HIV, but rather as a conceptualization that HIV/AIDS is part of the modern world where traditional behavioural standards are no longer formative and where indigenous or subsistence modes of life have been replaced by external ones. The reality is that Namibians view HIV/AIDS as a component of the social process of modernization.

There were also metaphors that equated persons with HIV/AIDS to sex, promiscuity, and death. The danger with some of these indigenous terminologies is that they help to sweep the HIV/AIDS epidemic under the carpet. This is so because they encourage stigma, discrimination, and rights abuses of people living with HIV/AIDS. Many derogatory expressions also subtract from efforts such as Voluntary Counseling and Testing (VCT) for HIV/AIDS, disclosure that one is HIV-positive, and starting or adhering to antiretroviral therapy.

On the other hand, the results of this study suggest that slang words and derogatory language towards HIV/AIDS may have helped students and teachers to form localized meanings of the epidemic. The language used to describe persons living with HIV and the symptoms that students associated with AIDS were part of the informal curriculum through which AIDS was understood. In certain cases, local terminologies that were associated with HIV/AIDS were meant to protect individuals by way of instilling fear. Sometimes local descriptions of HIV/AIDS were meant to convey subtle messages, for example, when referring to a person perceived to have been infected with the virus.

The use of certain metaphors very often reflected the students' worldviews of HIV/AIDS. We believe that some of the current efforts to reduce stigma against people living with HIV/AIDS and interventions to prevent and manage HIV/AIDS (ABC-Abstinence, Be faithful, Condoms; antiretroviral therapy) may fail if they are not anchored on the local people's IKS. Predominant terms should be included into variegated regional HIV/AIDS education curricula: Caprivi (*simbandembande*), Kavango (*kambumburu*), Khomas (*ekiya, ekomba, four letters*), Ohangwena (*okakiya*, killer disease, *four letters*, modern disease), Omusati (*kadhipagi*), and Oshikoto (*okakwega, oondanda ne*).

Correct perceptions of HIV/AIDS symptoms may be life-saving because individuals can avoid risky sexual behaviours with those that are infected. In this study, HIV/AIDS symptoms were perceived through more typical and traditional lenses of body deterioration like wasting, opportunistic infections, and weakness. Some of the novel symptoms seemed to be a product of long-term use of antiretroviral therapy (e.g. abnormal fat distribution due to lipodystrophy). Yet, there were also novel descriptions of HIV/AIDS symptoms such as big head, red eyes, stiff neck, painful joints, red lips, change of skin colour, and impaired vision. Besides physical body symptoms, psychological manifestations of HIV/AIDS (e.g. anger, unfriendliness, withdrawal, and stress) were also documented. The results also suggest that apart from body symptoms of disease, indigenous experiences of HIV/AIDS were now in transit to more psychological manifestations such as stress and depression.

The symptoms noted in this study, including the terms used for AIDS and people with AIDS, were essentially the same in all six regions, thus emphasizing that the etiological and biological reality of AIDS is quite consistent from region to region. However, the cultural constructions of HIV/AIDS differed by region. This revelation has implications for AIDS interventions because while the curriculum for the biology of HIV/AIDS may be the same across regions, the inclusion into the curriculum of cultural schemas and lenses through which students understand AIDS need to be variegated.

## Conclusions and recommendations

This study attempted to determine the students' knowledge of the indigenous names, meanings, and caricatures of HIV/AIDS. Symptoms which indigenous people associated with HIV/AIDS were also revealed. Once imbued into the formal school curricula, such indigenous vocabularies may help teachers and students to find local meanings that resonate with their easy-to-understand social constructions of the HIV/AIDS epidemic. We recommend that HIV/AIDS interventions targeting students should be anchored on their IK of the disease. Further, public awareness campaigns should be conducted in order to reduce the use of indigenous caricatures of HIV/AIDS that exacerbate stigma, embarrassment, discrimination, and human rights abuses. This will help change people's attitudes and lead to increased uptake of VCT, disclosure, and improved adherence to antiretroviral therapy.

## Competing interests

The authors declare that they have no competing interests.

## Authors' contributions

KCC participated in the conceptualization of the study, collected data, conducted the analysis, and wrote the manuscript. CDK, CNSS, and DZ participated in the conceptualization of the study, supervised the study, and made critical comments on the draft manuscript. CNSS also conducted Oshiwambo to English translations. All authors read and approved the final manuscript.
